# Degenerative Nucleus Pulposus Cells Derived Exosomes Promoted Cartilage Endplate Cells Apoptosis and Aggravated Intervertebral Disc Degeneration 

**DOI:** 10.3389/fmolb.2022.835976

**Published:** 2022-03-14

**Authors:** Xiaofei Feng, Yongchao Li, Qihang Su, Jun Tan

**Affiliations:** ^1^ School of Medicine, Tongji University, Shanghai, China; ^2^ Department of Orthopedics, Shanghai Tenth People’s Hospital, Shanghai, China; ^3^ Department of Spinal Surgery, Shanghai East Hospital, Shanghai, China

**Keywords:** nucleus pulposus, exosome, cartilage endplate, intervertebral disc degeneration, apoptosis

## Abstract

Intervertebral disc (IVD) degeneration is a complex multifactorial disease model, which pathogenesis has not been fully defined. There are few studies on the information interaction between nucleus pulposus (NP) cells and cartilage endplate (CEP) cells. Exosomes, as a carrier of information communication between cells, have become a research hotspot recently. The purpose of this study was to explore whether degenerative NP cells-derived exosomes promoted CEP cells apoptosis and aggravated IVD degeneration. The degenerative NP cells model was induced by TNFα. NPC exosomes were isolated from the supernatant of the NP cell culture medium. The viability of NP cells and CEP cells was examined by CCK-8 assays. The exosomes were identified by TEM, NTA, and western blot. Extracellular matrix (ECM) metabolism was measured by cellular immunofluorescence and qRT-PCR. Apoptosis was detected by flow cytometry and TUNEL. X-ray and magnetic resonance imaging (MRI), as well as hematoxylin-eosin (H&E), Safranine O-Green staining was adopted to evaluate IVD degeneration grades. TNFα had a minor impact on NPC viability but inhibited ECM synthesis and promoted ECM degradation. TNFα-NPC-Exo had less effect on CEPC proliferation but promoted CEPC apoptosis and affect ECM metabolism, inhibiting aggrecan and collagen II expression and enhancing MMP-3 expression. TNFα-NPC-Exo aggravates IVD degeneration in a rat model and promoted CEPC apoptosis. In conclusion, this study demonstrated that degenerated NPC-exosome could induce apoptosis of CEPCs, inhibit ECM synthesis, and promote ECM degradation. In addition, it was proved that degenerated NPC-exosome aggravates IVD degeneration.

## Introduction

Low back pain (LBP) is a common global healthcare challenge leading to more disability than any other medical problem in the world in recent decades (Hoy et al., 2014; Hall et al., 2019; Vos et al., 2020). It is estimated that up to 84% of adults have LBP at some time in their lives, while 10% are chronically disabled (Hartvigsen et al., 2018). LBP has been closely associated with t intervertebral disc (IVD) degeneration, a special soft tissue between the vertebrae that distributes and absorbs applied loads and lends flexibility to the spine (Roberts et al., 2006; Khan et al., 2017). IVD is composed of annulus fibrosus (AF), nucleus pulposus (NP), and cartilage endplate (CEP) (Raj 2008). The NP, a white hydrated gelatinous tissue, is the main functional component of the IVD to resist compressive loads and reversible deformation. NP consists of nucleus pulposus cells (NPCs) and extracellular matrix (ECM). As for the ECM, two major components are extremely important for its integrity: water-binding proteoglycans such as aggrecan and the type I and type II collagen network that provides tensile strength (Roughley 2004). CEP is similar to articular cartilage which consists of cartilage endplate cells (CEPCs) and type II collagen. It connects the vertebral body to the IVD and has many tiny cavity gaps in the tissue that plays an important role in the movement of fluid and solutes in and out of the IVD (Chen et al., 2017). After nearly 50 years of in-depth research, IVD degeneration is considered to be a complex and multifactorial integrated disease model. IVD degeneration is a pathological process that consists of an inflammatory response, cell loss, and degradation of the extracellular matrix under the stimulation of major pathogenic factors, such as oxidative stress, DNA damage, pro-inflammatory factors, nutritional deficiency, and abnormal mechanical load (Hadjipavlou et al., 2008), but its pathogenesis has not been fully defined. Based on anatomy, it is believed that the main function of CEP is to provide nutrition for NP (Roberts et al., 2006). However, the communication between NP cells and CEP cells has not been studied.

As a new carrier of information communication between cells, exosomes can be secreted by almost all living cells. Exosomes contain nucleic acids, proteins, lipids, and other biological molecules and maintain stable activity, which can be absorbed by recipient cells to play a variety of biological functions (Mathieu et al., 2019; Wortzel et al., 2019). So far, relevant studies have shown that exosomes are involved in basic physiological functions such as neuronal communication, antigen presentation, immune response, organ development, cancer progression, coronary heart disease, inflammation, and viral infections (Raposo et al., 1996; Skog et al., 2008; Korkut et al., 2009; Kadiu et al., 2012; Frühbeis et al., 2013; Kulshreshtha et al., 2013; Robbins and Morelli 2014; Ailawadi et al., 2015). In the study of exosomes in IVD degeneration, most attempts are being made to protect IVD from stem cell exosomes (Lu et al., 2017; Hu et al., 2020).

Therefore, in this study, it is assumed that exosomes secreted by NP cells reach the CEP tissue through osmosis, are taken up by CEP cells, and affect the degeneration process of CEP. Subsequently, a series of *in vivo* and *in vitro* experiments were conducted to explore the role and mechanism of NP cells derived exosome in the apoptosis of CEP cells and IVD degeneration. We found that degenerated NPC-exosome could induce apoptosis of CEPCs, inhibit the synthesis of ECM and promote degradation of ECM. Further study showed that degenerated NPC-exosome aggravates IVD degeneration.

## Materials and Methods

### Cell Isolation and Culture

Human NP tissues and CEP tissues were obtained from 12 separate patients who underwent spine surgery because of burst thoracolumbar fracture (Table 1). Patient inclusion criteria: age 20–40 and IVD in MRI Pfirrmann grading grade I-II. Exclusion criteria: age younger than 20 years or older than 40 years, patients with chronic disc herniation, lumbar spondylolisthesis and other spinal diseases, Pfirrmann grade Ⅲ-V, suffers from other systemic diseases. MRI Pfirrmann grade is graded according to the water content of the intervertebral disc and the height of the intervertebral space. Grade Ⅲ or higher indicates degeneration of the disc. The experimental protocol was approved by the Ethics Committee of the East Hospital affiliated whit Tongji University with informed consent from the patients. According to the morphological difference of the tissues, NP tissues and CEP tissues were separated from IVD tissues by microscopy. The NP tissues and CEP tissues were treated with 0.25% trypsin (Sigma, United States) for 1 h at 37°C and by 0.2% type II collagenase (Sigma, United States) digestion for 4 h at 37°C. The digest underwent centrifugation and filtration by a 70 μm pore size mesh (Falcon, United States). Then NPCs and CEPCs were collected and cultured in a complete Dulbecco’s modified Eagle’s medium (DMEM, South Logan, United States)/F12 medium supplement with 20% fetal bovine serum (FBS, Grand Island, United States), 1% penicillin-streptomycin (Grand Island, United States) in T25 flasks (Corning, United States) under the following conditions: 5% CO_2_ and at 37°C in a humidified incubator (Thermo Fisher Scientific, United States). When grew to confluence, the cells were digested by 0.25% trypsin/0.05% EDTA and passed into T75 flasks (Corning, United States) for culture. The isolation and culture of CEPCs are similar to NPCs. The NPCs and CEPCs from passage 3 were plated into experimental plates for all of the experiments.

**TABLE 1:** The NPCs and CEPCs of patients selected for the study.

Patient ID	Age	Sex	Fracture section	NP tissues	CEPs tissues
1	24	Male	L1	T12/L1, L1/L2	T12, L2
2	37	Female	L1	T12/L1, L1/L2	T12, L2
3	29	Male	L2	L1/L2, L2/L3	L1, L3
4	28	Male	T12	T11/T12, T12/L1	T11, L1
5	31	Female	L1	T12/L1, L1/L2	T12, L2
6	25	Female	L1	T12/L1, L1/L2	T12, L2
7	32	Male	L2	L1/L2, L2/L3	L1, L3
8	24	Female	L1	T12/L1, L1/L2	T12, L2
9	29	Male	L2	L1/L2, L2/L3	L1, L3
10	33	Female	L2	L1/L2, L2/L3	L1, L3
11	25	Male	L1	T12/L1, L1/L2	T12, L2
12	28	Male	L2	L1/L2, L2/L3	L1, L3

NPCs, nucleus pulposus cells; CEPCs, cartilage endplate cells; T, thoracic vertebral body; L, lumbar vertebral body.

### Cell Viability Assay

According to the instructions of the manufacturer, CCK-8 assays (Beyotime Biotechnology, China) were performed to detect the viability of NPCs and CEPCs. NPCs were cultured in 96-well plates at a density of 2 × 10^4^ cells/well. NPCs were treated with 0, 1, 5, 10, 20, 30, 50 ng/ml TNFα (Sigma-Aldrich, United States) for 24 h to determine the appropriate TNFα treatment concentration. And CEPCs were treated with 0, 10, 20, 30, 40, 50 μg/ml Norm-NPC-Exo and TNFα-NPC-Exo for 24 h to determine the suitable NPCs exosome treatment concentration. In addition, CEPCs were treated with 20 μg/ml exosomes for 2, 4, 6, 8, 10, 12, and 14 days to calculate CEPC proliferation multiple. Subsequently, each well was added to 10 μl CCK-8 solution and 90 μl culture media and incubated for 4 h at 37°C. Finally, an auto-microplate reader was used to read the absorbance of each well. The cell viability was calculated by the formula which is the ratio of absorbance of sample to the absorbance of untreated control and treated.

### Exosome Isolation

NPC-exosomes isolation procedures were performed by differential Overspeed centrifugation. The NPCs were removed from the original culture medium and replaced with a new exosome-free culture medium (South Logan, United States) for 48 h. The culture supernatant was evenly divided into a 50 ml centrifuge tube (Coming, United States) and then suspended to 40 ml with phosphate-buffered saline (PBS, South Logan, United States). After balancing, the experimental samples were centrifuged at 300 g for 10 min at 4°C to eliminate cells, at 2,000 g for 20 min at 4°C to obtain the apoptotic body, at 10,000 g for 30 min at 4°C to obtain microvesicles and at 10,000 g for 70 min at 4°C to obtain exosomes. At each step, the supernatant was transferred to new centrifuge tubes and the pellets were immediately resuspended in PBS. Exosomes were collected for experiments or stored in the refrigerator at −80°C.

### Exosome Characterization

At present, internationally recognized methods for exosome identification must include observation of exosome size and morphology by electron microscope, detection of exosome particle diameter distribution by nanoparticle tracking analysis, and detection of exosome marker proteins by western blot. The extracted exosome samples were removed from the refrigerator at −80°C and placed in an icebox. After dissolving, 15 μl exosome samples were absorbed with a pipette gun and placed on a copper wire for 1 min. The filter paper was used to blot the exosome samples on the copper wire, and then 15 ul of 2% uranium deoxy acetate staining solution (Sigma, United States) was used to stain at room temperature for 1 min. Then, the filter paper was used to blot the exosome samples on the copper net, and the dyed samples were placed under the lamp for 10 min to observe and take photos by transmission electron microscope (TEM, Philips Tecnai, Germany). The grids were examined using the TEM at 80 kV. NPC-derived particles were resuspended and further diluted in 1 ml PBS to analyze their number and size distribution using the ZetaView Particle Metrix (PMX, Germany) according to the handbook. The extracted exosome samples were detected based on the marker expression of exosomes (TSG101, CD9, and CD63, Abcam, United Kingdom) and nuclear marker protein (Calnexin, Abcam, United Kingdom).

### Exosome Uptake by Cartilage Endplates

Purified Norm-NPC-Exo and TNFα-NPC-Exo were incubated with PKH26 (Sigma, United States) for 5 min at room temperature. After being washed thrice in PBS with 120,000 g centrifugation for 60min, the marked exosomes were suspended in basal medium and incubated with CEPCs for 6 and 24 h at 37°C. CEPCs were washed thrice with PBS and stained the nuclei by4’,6-diamidino-2-phenylindole (DAPI; Vector Labs, United States). The stained cells were observed and taken photos under the fluorescence microscope (Leica, Germany).

### Immunofluorescence

NPCs were cultured in 24-well plates (4 × 10^4^ cells/well), treated with 5 ng/ml TNFα for 24 h. Similarly, CEPCs were cultured in 96-well plates (4 × 10^4^ cells/well), treated with 20 μg/ml Norm-NPC-Exo and TNFα-NPC-Exo for 24 h. Cells were fixed with fresh 4% paraformaldehyde (Beyotime Biotechnology, China) for 20 min and washing with PBS contained 0.1% Tween-20(Sigma, United States) in the following. The cells were incubated with 0.2% Triton X-100 (Beyotime Biotechnology, China) for 15 min. Then the cells were blocked with 5% goat serum for 60 min and treated with the primary antibody against aggrecan (1:50), collagen II (1:100), and MMP-3 (1:100) (Abcam, United Kingdom)overnight at 4°C, followed by incubation with FITC-conjugated (Cell Signaling Technology, United States)secondary antibodies for 1 h at 37°C. The fluorescent images were obtained by fluorescence microscope (Leica, Germany) and the intensity was quantified by the ImagePro Plus software (Version 6.0, Media Cybernetics, United States). The images captured for Immunofluorescence used ×40 objective and Z stack depth optimization. About 100 cells were chosen for semi-quantitative analysis of Aggrecan, Collagen II, and MMP-3 fluorescence intensity.

### RNA Extraction and Quantification

Total RNAs from cells and tissues were extracted using TRIzol and TRIzolLS Reagent (Life Technologies). Respectively, RNA expression levels were determined using NanoDrop 2000 system (Thermo Scientific, United States). Specific mRNA primers were synthesized by GenePharma. RNA was reverse-transcribed using PrimeScipt RT Reagent Kit (Takara, Japan). 40 cycles were used for qPCR. mRNA levels were determined using real-time analysis with SYBR Green on a StepOne-Plus machine (Applied Biosystems, United States). Relative expression levels of the mRNAs in cells and tissues were normalized to GAPDH. Fold changes in expression were calculated by a comparative threshold cycle (Ct) method using formula 2^-(△△ct)^. The primers used were listed in Supplementary Table S1.

### Flow Cytometry Analysis

CEPCs were plated into 24-well plates at a density of 5 ×10^5^ cells/well. To test the apoptosis on cells, CEPCs were treated with 20 μg/ml Norm-NPC-Exo and TNFα-NPC-Exo for 24 and 48 h. Cells kept in the normal medium served as controls. Subsequent treatment, CEPCs apoptosis rates were evaluated by flow cytometry using an Annexin V/PI apoptosis detection kit (Beyotime Biotechnology, China). CEPCs were washed once with PBS, centrifuged at 500 g for 5 min, removed supernatant, resuspended in 195 μl binding buffer, and incubated with 5 μl FITC-Annexin V and 10 μl PI for 15 min at room temperature. Staining cells were analyzed using the FACS can flow cytometry system (Becton Dickinson, United States).

### Western Blot Analysis

All protein expression levels were determined by western blot analysis. The total protein of cells and exosomes was extracted using the BCA protein extraction kit (Beyotime Biotechnology, China). The protein samples were added to the loading buffer and boiled for 5 min. Following treatment, the proteins were separated using sodium dodecyl sulfate-polyacrylamide (SDS) gels (12%) by polyacrylamide gel electrophoresis (PAGE, Beyotime Biotechnology, China). The proteins were transferred to polyvinylidene fluoride membranes (PVDF membranes, Millipore, United States) using a wet blotting method. Following blocking with 5% non-fat milk in Tris-buffered saline containing 0.1% Tween-20 (TBST) for 1 h. The membranes were incubated overnight at 4°C with suitable different primary antibodies. And after being washed 3 times in TBST, the membranes were incubated with the secondary antibodies for 2 h at room temperature. After adding ECL luminescence solution, the detection of the band was performed using the Li-Cor Odyssey 9120 Infrared Imaging System (Bio-Rad, United States). The intensity of bands was quantified by the ImagePro Plus software (Version 6.0, Media Cybernetics, United States). The antibodies used are as follows: Calnexin (1:1,000, Abcam, United Kingdom), CD9 (1:1,000, Abcam, United Kingdom), CD63 (1:1,000, Abcam, United Kingdom), TSG101 (1:1,000, Abcam, United Kingdom), caspase-3 (1:800; CST, United States) and cleaved caspase-3 (1:800; CST, United States), Bcl-2 (1:800; CST, United States), Bax (1:800; CST, United States). The antibody for GAPDH (1:2000; Abcam, United Kingdom) was used as a control.

### Needle Puncture Rat Model of Intervertebral Disc Degeneration

The animal experiment was approved by the Animal Care and Use Committee of East Hospital affiliated with Tongji University, and the related management, operation, treatments, and animal care standards were followed. The experimental animals were purchased from Shanghai Slack Animal Co. Ltd. The strain was SD rats, female, aged 6 weeks. The molding procedure (Zhang et al., 2009) was as follows. The rat was anesthetized with 4% chloral hydrate (Sangon Biotech, Shanghai, China), 10 ml/kg by intraperitoneal injection. After the rat coccygeal disc was determined, the discs were punctured with a 28-gauge disinfected needle in a parallel direction to the CEP towards the NP from the four avascular zones through the skin and ligament, held for 30 s. Intervertebral disc puncture was performed by spacer puncture, and the no-puncture segments were used as self-controls. 32 rats underwent the surgery while 8 rats underwent no surgical intervention as negative controls. After the initial operation for 1 week, the rats were randomly divided into 4 groups (non-injection, normal saline, Norm-NPC-Exo, and TNFα-NPC-Exo group) with 8 rats in each group. Subsequently, A total of 2 μl sterile NS containing different purified exosomes (approximately 2 × 10^6^ particles) were slowly injected into the punctured discs using a microlitre syringe.

### Radiography and Magnetic Resonance Imaging Examination

Mammography X-rays were taken at 2, 4, and 6 weeks after modeling and injection. The rats were injected intraperitoneally with 10% chloral hydrate 10 ml/1 kg. After successful anesthesia, the limbs of rats were fixed for imaging examination. The X-ray films were obtained on an X-ray system (United Imaging, China) and used to assess disc height. The Disc Height Index (DHI) was used to evaluate disc height loss after modeling according to the method as previously described (Masuda et al., 2005). According to the Pfirrmann classification (Pfirrmann et al., 2001) (Supplementary Table S2), MRI films were performed on an MRI system (United Imaging, China) to evaluate disc degeneration grades, which ranged from grade I to grade IV.

### Histological Evaluation and *in situ* Apoptosis Detection

After the imaging examination, all rats were sacrificed by intraperitoneal administration of overdose pentobarbital sodium. The tail of the rat was dissected quickly. Skin, ligament, and other accessories were removed and the vertebral-disc-vertebral complex of the corresponding segment was obtained to fixed in 4% paraformaldehyde. Then tissues were decalcified, dehydrated, cleared with dimethyl benzene, and embedded in paraffin. The embedded specimens were fixed in the slicer (Leica embedder, Buffalo Grove, IL) and sliced consecutively with a thickness of 5 μm. The sections were stained with Hematoxylin-Eosin and Safranin O. Histological images were obtained by the light microscopy mode of a fluorescence microscope (Leica, Germany) under a 20×, ×50, and ×200 magnification. Histologic analysis of intervertebral discs based on Pfirrmann grade (Che et al., 2019). Likewise, the sections were stained with terminal deoxynucleotidyl transferase (TdT)-mediated dUTP nick end labeling (TUNEL). The apoptotic rate was detected using the *In Situ* Cell Death Detection Kit Fluorescein (Roche, Germany) according to the instruction book. TUNEL images were obtained using filters for fluorescein isothiocyanate (FITC) and DAPI. TUNEL-positive cells were counted in three fields in the CEP region using the Image-Pro Plus software (Version 6.0, Media Cybernetics, United States) for semi-quantitative analysis.

### Statistical Analysis

All numerical data are expressed as the mean ± standard deviation (SD) for at least three separate experiments. Statistical analysis was performed using the Statistical Product and Service Solutions 21.0 software (SPSS Inc., Chicago, United States) or Prism software (GraphPad Prism, version 8.0, United States). The data by a normal distribution and equal variances was performed by unpaired two-tailed Student’s *t*-test between two groups. The data following unequal variances was performed by Welch *t*-test and following non-normal distribution was performed by Mann–Whitney *U*-test. Multiple group comparisons were performed by one-way analysis of variance (ANOVA) (normal distribution) or Kruskal–Wallis (non-normal distribution) test between groups. *p* < 0.05 were considered statistically different.

## Results

### TNFα had a Minor Impact on Nucleus Pulposus Cells Viability but Inhibited Extracellular Matrix Synthesis and Promoted Extracellular Matrix Degradation

**FIGURE 1:**
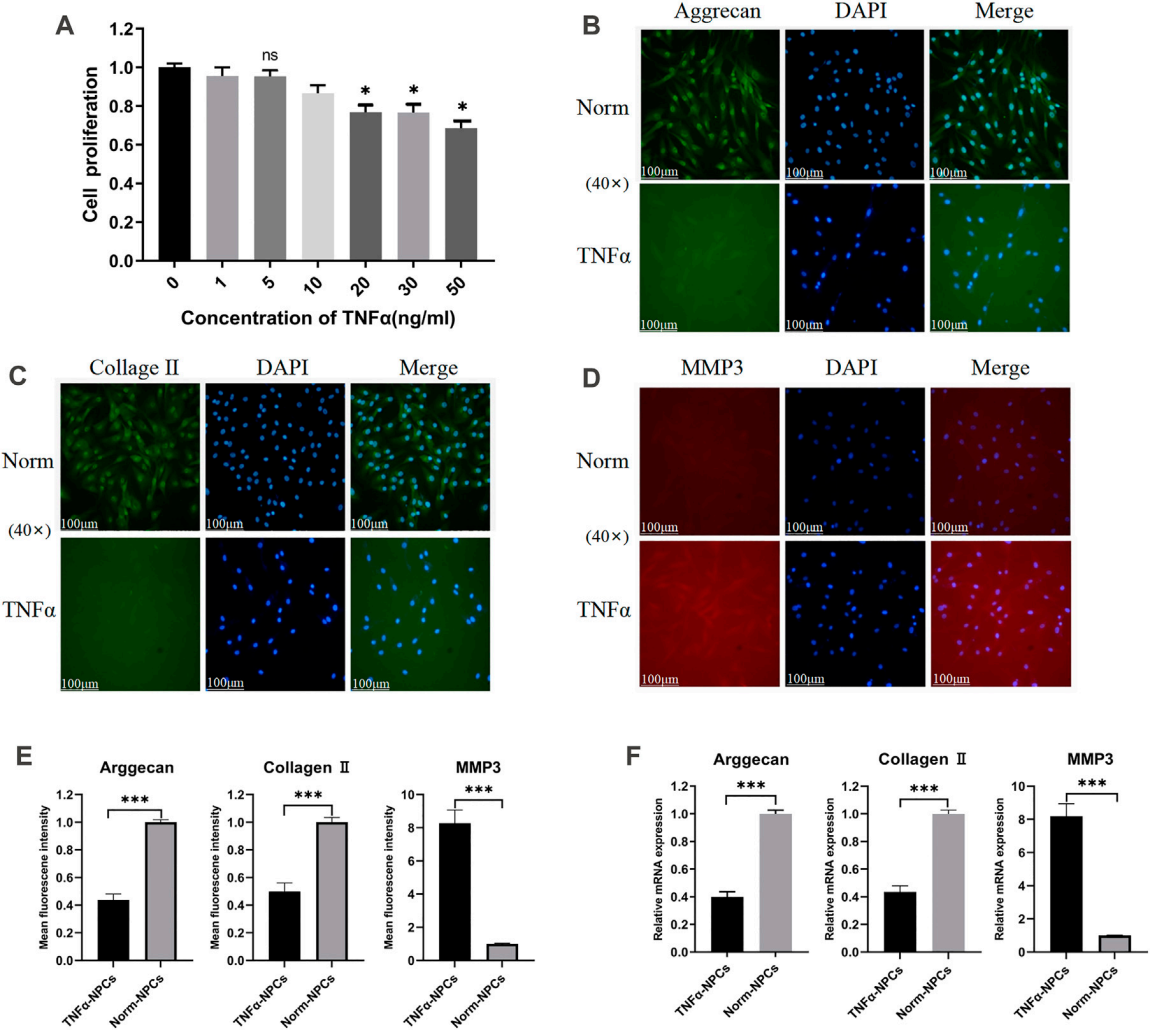
Effect of TNFα on the viability and ECM metabolism of NPCs. **(A)** NPCs were treated with 0, 1, 5, 10, 20, 30, 50 ng/ml TNFα for 24 h and detected by CCK-8 assay (*n* = 3). **(B)** Typical images of immunofluorescence of Aggrecan in NPCs photographed by fluorescence microscopy (scale bar = 100 μm). **(C)** Typical images of immunofluorescence of Collagen II in NPCs photographed by fluorescence microscopy (scale bar = 100 μm). **(D)** Typical images of immunofluorescence of MMP3 in NPCs photographed by fluorescence microscopy (scale bar = 100 μm). **(E)** Semi-quantitative analysis of Aggrecan, Collagen II, and MMP-3 fluorescence intensity (cells = 100). **(F)** The expression levels of Aggrecan, Collagen II, and MMP-3 were analyzed by qRT-PCR in NPCs, which were cocultured for 24 h with 5 ng/ml TNFα. All results are representative of at least three independent experiments and each value is the mean ± s.d. of three determinations. *p < 0.05, **p < 0.01 and ***p < 0.001.

NPCs were treated with 0, 1, 5, 10, 20, 30, 50 ng/ml TNFα for 24 h, and then the viability of the cells was detected by the CCK-8 assay. The results showed that when the concentration of TNFα was inferior to 5 ng/ml, the influence on NPC viability was minimal. when the concentration was above 20 ng/ml, the viability of the cells was decreased by approximately 25% (Figure 1A). Hence, 5 ng/ml TNFα was selected to incubate NPCs for 24 h in the following experiments. The primary function of NPCs is to synthesize and secrete two major components of the ECM: aggrecan and collagen II. The main components of ECM degradation are matrix metalloproteinase (MMP) enzymes and a disintegrin and metalloproteinase with thrombospondin motifs (ADAMTS), mainly including MMP-1,3,7,9,13 and ADAMTS-4,5,9 (Hayes et al., 2001; Mizrahi et al., 2013; Vo et al., 2013; Dagistan et al., 2015). When NPCs were treated with 5 ng/ml TNFα for 24 h, the expression of aggrecan and collagen II was significantly decreased (Figures 1B,C), while the expression of MMP3 was significantly increased (Figure 1D) by immunofluorescence assays and qRT-PCR (Figures 1E,F).

### Characterization of Nucleus Pulposus Cells Exosomes and Uptake of Exosomes by Cartilage Endplate Cells

**FIGURE 2:**
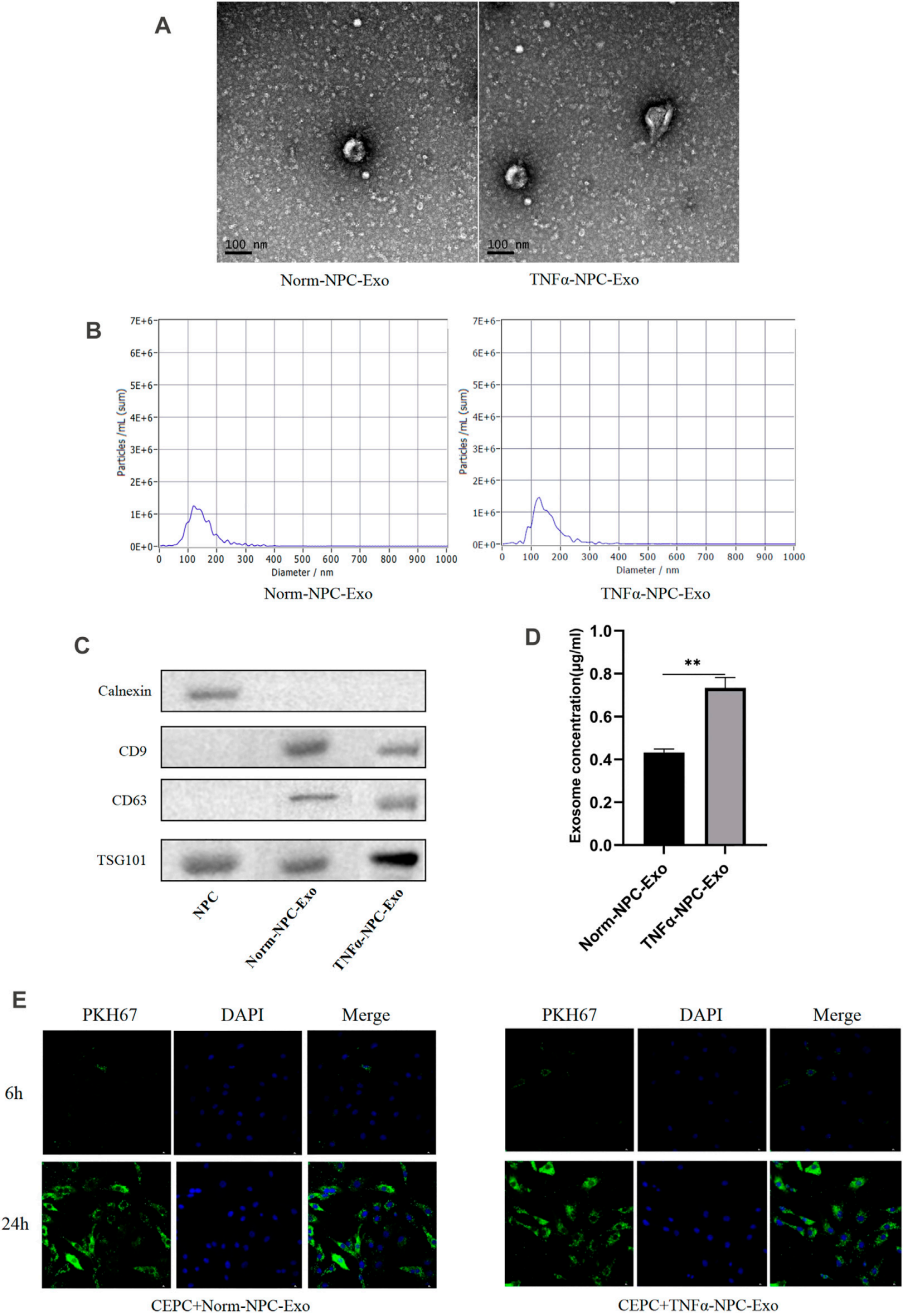
Identification and characterization of nucleus pulposus-derived exosomes and the uptake of exosomes in CEPCs. **(A)** Transmission electron micrograph of purified particles. The image showed small vesicles of approximately 100 nm in diameter (scale bar = 100 nm). **(B)** Size distribution of vesicles secreted by NPCs determined by NTA. The average particle size was 153.6 nm. **(C)** Expression of exosomes markers (CD9, CD63, and TSG101) and nuclear marker (Calnexin) detected by Western blot. The protein expression of exosomes markers was detectable in NPC exosomes but not NPCs. **(D)** NPCs were cultured under normal medium and 5 ng/ml TNFα conditions for 24 h, and the protein expression of exosomes was measured by immunoblot. TNFα increased the production of exosomes in NPCs. **(E)** The uptake of NPC exosome in CEPC. Compared with co-culture for 6 h, a large number of NPC exosomes were taken up in CEPCs after co-culture for 24 h. All results are representative of at least three independent experiments and each value is the mean ± s.d. of three determinations. *p < 0.05, **p < 0.01 and ***p < 0.001.

According to the characteristics of exosomes, the morphology and phenotypes of isolated exosomes were identified. We isolated, characterized and quantified exosomes from NPCs under norm and TNFα conditions by using transmission electron microscopy (TEM), nanoparticle tracking analysis, and immunoblot (using CD9, CD63, TSG101, and Calnexin as markers). The morphology of isolated exosomes showed a double concave disc shape under TEM (Figure 2A). The peak particles size of NPC-derived exosomes was 126.0 nm, and the peak area accounted for 91.3%. The measured average particle size was 153.6 nm, which was consistent with the distribution range of exosomes ranging from 30 to 200 nm (Figure 2B). The protein expression of exosomes markers (CD9, CD63, and TSG101) were all detectable in the particles, and the marker protein of the nucleus (calnexin) was undetectable (Figure 2C). The above detection methods identified these isolated particles as NPC-derived exosomes. Total exosomes proteins normalized by cell number were significantly increased when NPCs were exposed to TNFα conditions (Figure 2D), which suggested that TNFα could promote the production of NPC exosomes. After incubated with CEPCs for 6 and 24 h, PKH26-labelled NPC exosomes remarkably showed blue fluorescence in the cytoplasm of CEPCs after 24 h (Figure 2E), indicating exosomes uptake by CEPCs.

### TNFα-Nucleus Pulposus Cells-Exo had Less Impact on Cartilage Endplate Cells Proliferation but Promoted Cartilage Endplate Cells Apoptosis and Affect Extracellular Matrix Metabolism

**FIGURE 3:**
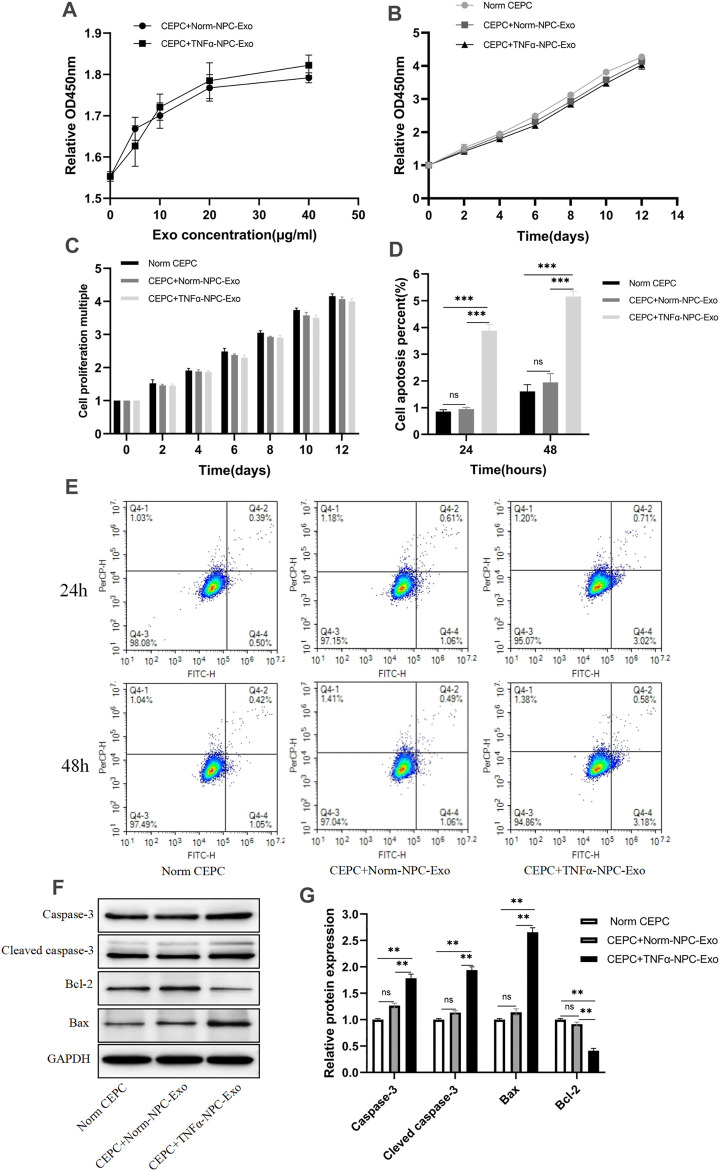
Effects of Norm-NPC-Exo and TNFα-NPC-Exo on proliferation and apoptosis of CEPCs. **(A)** CEPCs were treated with 0, 10, 20, 30, 40, 50 μg/ml Norm-NPC-Exo and TNFα-NPC-Exo for 24 h and detected by CCK-8 assay (*n* = 3). **(B)** CEPCs were treated with 20nμg/ml Norm-NPC-Exo and TNFα-NPC-Exo for 0, 2, 4, 6, 8, 10, 12 days and detected by CCK-8 assay (*n* = 3). **(C)** The cell viability of different groups of CEPC was calculated according to OD value. The results showed that there was no significant difference in the cell proliferation multiple among the three groups. **(D)** The percentage of apoptotic CEPCs could be increased to 3.88 ± 0.05% at 24 h or to 5.16 ± 0.04% at 48 h on TNFα-NPC-Exo treated CEPCs. Compared with the control and Norm-NPC-Exo group, TNFα-NPC-Exo causes a significant change in apoptosis rate. **(E)** CEPCs were treated with Normal medium, Norm-NPC-Exo, and TNFα-NPC-Exo for 24 and 48 h. Representative dot plots of apoptosis flow cytometry detection were shown. **(F)** Western blot analyzed caspase-3, cleaved caspase-3, Bax, and Bcl-2 in CEPCs after treatment of different exosomes. **(G)** Semi-quantitative analysis of caspase-3, cleaved caspase-3, Bax, and Bcl-2 levels (*n* = 3). All results are representative of at least three independent experiments and each value is the mean ± s.d. of three determinations. *p < 0.05, **p < 0.01.

CEPCs were treated with 0, 10, 20, 30, 40, 50 μg/ml Norm-NPC-Exo and TNFα-NPC-Exo for 24 h. Then the viability of the cells was detected by the CCK-8 assay. The results showed that different concentrations of Norm-NPC-Exo and TNFα-NPC-Exo had little impact on the proliferation of CEPC. Especially, which the concentration of Norm-NPC-Exo and TNFα-NPC-Exo exceeds 20 μg/ml, the influence on CEPC viability was less (Figure 3A). Therefore, 20 μg/ml Norm-NPC-Exo and TNFα-NPC-Exo were selected to incubate CEPCs in the following experiments. Accordingly, CEPCs were treated with 20 μg/ml exosomes for 2,4,6,8,10,12 and 14 days, and the viability of the cells was detected by the CCK-8 assay. The results identified that the growth trend of the three groups was almost similar (Figure 3B), and there was no significant difference in the proliferation rate (Figure 3C). Apoptosis of CEPCs treated with 20 μg/ml Norm-NPC-Exo and TNFα-NPC-Exo for 24 and 48 h was measured using flow cytometry. And activation of apoptotic pathways was detected by assessment of expression of apoptosis marker protein (Caspase-3, Cleaved caspase-3 Bcl-2, and Bax) using Western blot. The results demonstrated that 20 μg/ml TNFα-NPC-Exo could induce CEPC apoptosis at 24 and 48 h, compared with the other two groups. But there was no significant difference in the apoptosis rate of CEPC between the 20 μg/ml Norm-NPC-Exo group and control group (Figures 3D,E). Analogously, the expression levels of caspase-3, cleaved caspase-3, and Bax in CEPC increased following 20 μg/ml TNFα-NPC-Exo treatment, and the expression levels of Bcl-2 in CEPC decreased (Figures 3F,G).

**FIGURE 4:**
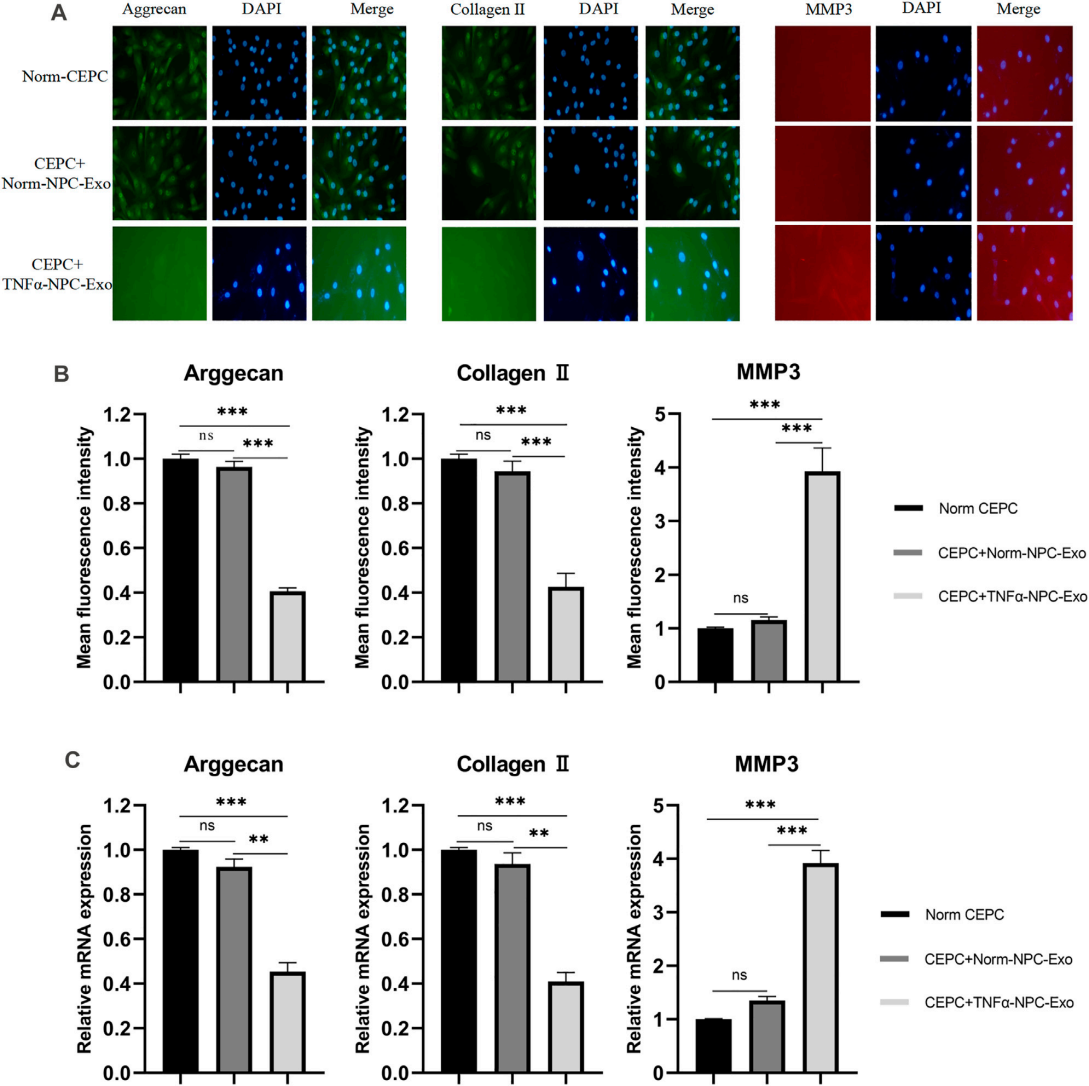
Effects of Norm-NPC-Exo and TNFα-NPC-Exo on ECM metabolism of CEPCs. **(A)** CEPCs were treated with 20 μg/ml Norm-NPC-Exo and TNFα-NPC-Exo for 24 h. Representative images of immunofluorescence of aggrecan, collagen II and MMP-3 in CEPCs photographed by fluorescence microscopy (scale bar = 100 μm). **(B)** Semi-quantitative analysis of aggrecan, collagen II, and MMP-3 fluorescence intensity (*n* = 3). Compared with the control and Norm-NPC-Exo group, the expression of aggrecan and collagen II in CEPC cytoplasm was significantly decreased at TNFα-NPC-Exo group, while the expression of MMP-3 was significantly increased. **(C)** The expression levels of aggrecan, collagen II and MMP-3 were analyzed by qRT-PCR in CEPCs. Aggrecan and collagen II expression were significantly decreased following TNFα-NPC-Exo treatment, while MMP-3 expression was significantly increased. All results are representative of at least three independent experiments and each value is the mean ± s.d. of three determinations. *p < 0.05, **p < 0.01 and ***p < 0.001.

The ECM of CEPCs was detected by immunofluorescence staining after being treated with normal complete medium, 20 μg/ml Norm-NPC-Exo and 20 μg/ml TNFα-NPC-Exo for 24 h. The results showed that the expression of aggrecan in CEPC cytoplasm was significantly decreased in the TNFα-NPC-Exo group. Similarly, the expression of collagen II was similar to aggrecan. As for MMP3, its expression increased significantly in CEPC cytoplasm (Figures 4A,B). Furthermore, qRT-PCR was used to detect the expression level of aggrecan, collagen II and MMP3 in CEPC cytoplasm among different groups. The results demonstrated that the expression of aggrecan and collagen II was significantly decreased, and MMP3 was high expression (Figure 4C).

### Intradiscal Injection of TNFα-Nucleus Pulposus Cells-Exo Aggravates Intervertebral Disc Degeneration in a Rat Model and Promoted Cartilage Endplate Cells Apoptosis

**FIGURE 5:**
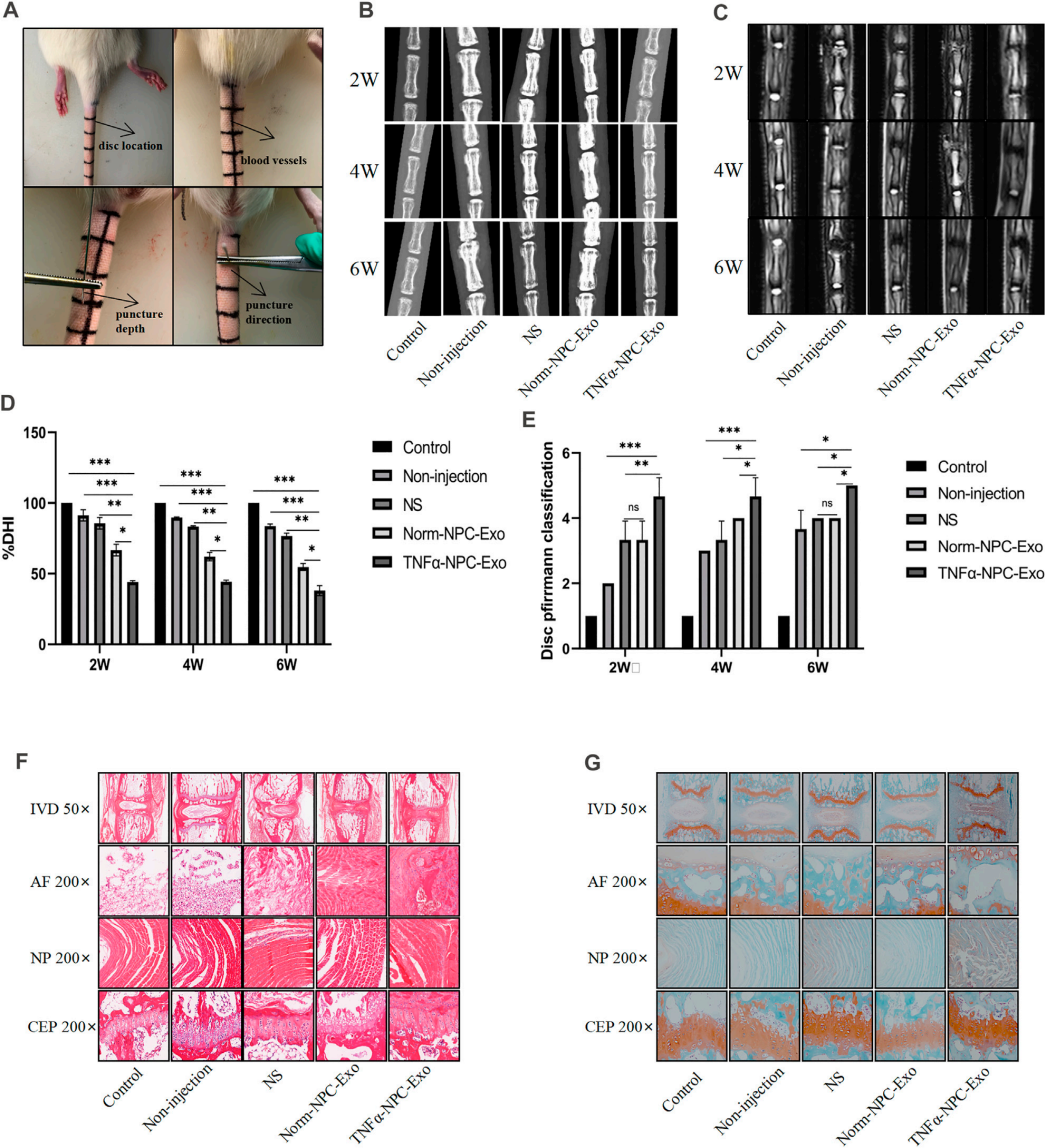
Intradiscal injection of TNFα-NPC-Exo alleviated the IVD degeneration in a rat model. **(A)** Modeling method of rat caudal vertebra by acupuncture. **(B)** Representative images of X-ray film were obtained at 2, 4, and 6 weeks after needle puncture. **(C)** Representative images of MRI film were obtained at 2, 4, and 6 weeks after acupuncture. **(D)** Changes in disc height index (DHI) of the indicated groups after a puncture. The DHI was measured at weeks 2, 4, 6 timing. A significant decrease of the DHI was observed in all puncture groups at 2 weeks after surgery. And the more serious decrease of the DHI was noted in TNFα-NPC-Exo groups (*n* = 8). **(E)** The change of MRI grade in the indicated groups after a puncture. The degree of disc degeneration by MRI grade was significantly higher in the TNFα-NPC-Exo groups than in the non-injection group (*n* = 8). **(F)** Representative images of HE staining. **(G)** Representative images of Safranine O-Green staining. Each value are expressed as mean ± s.d *p < 0.05, **p < 0.01, and ***p < 0.001.

We successfully established a rat caudal vertebra model of IVD degeneration by the needle puncture using a 28-gauge fine needle (Figure 5A). A total of 5 groups were prepared in vivo-study. These were the following: the control group, the non-injection group, the normal saline (NS) group, the Norm-NPC-Exo group, and the TNFα-NPC-Exo group. At 1, 3, and 5 weeks after the puncture, the exosome was injected into the punctured IVD. And the rat tails were examined at the 2, 4, 6 weeks periods by X-ray mammography and MRI imaging to evaluate disc height index (DHI) and grade of disc degeneration according to the Pfirrmann classification. In the rat IVD model, puncture segments showed that intervertebral space height decreased and endplate boundary defect at the 2 weeks. Intradiscal injection of NS, Norm-NPC-Exo, and TNFα-NPC-Exo demonstrated that the IVD exhibited more height loss and endplate defect at 2 weeks. Among them, there is a significant difference in the DHI was noted between the TNFα-NPC-Exo groups and other groups. Moreover, the loss of DHI and the grade of disc degeneration become more serious at 4 and 6 weeks in the TNFα-NPC-Exo groups.

**FIGURE 6:**
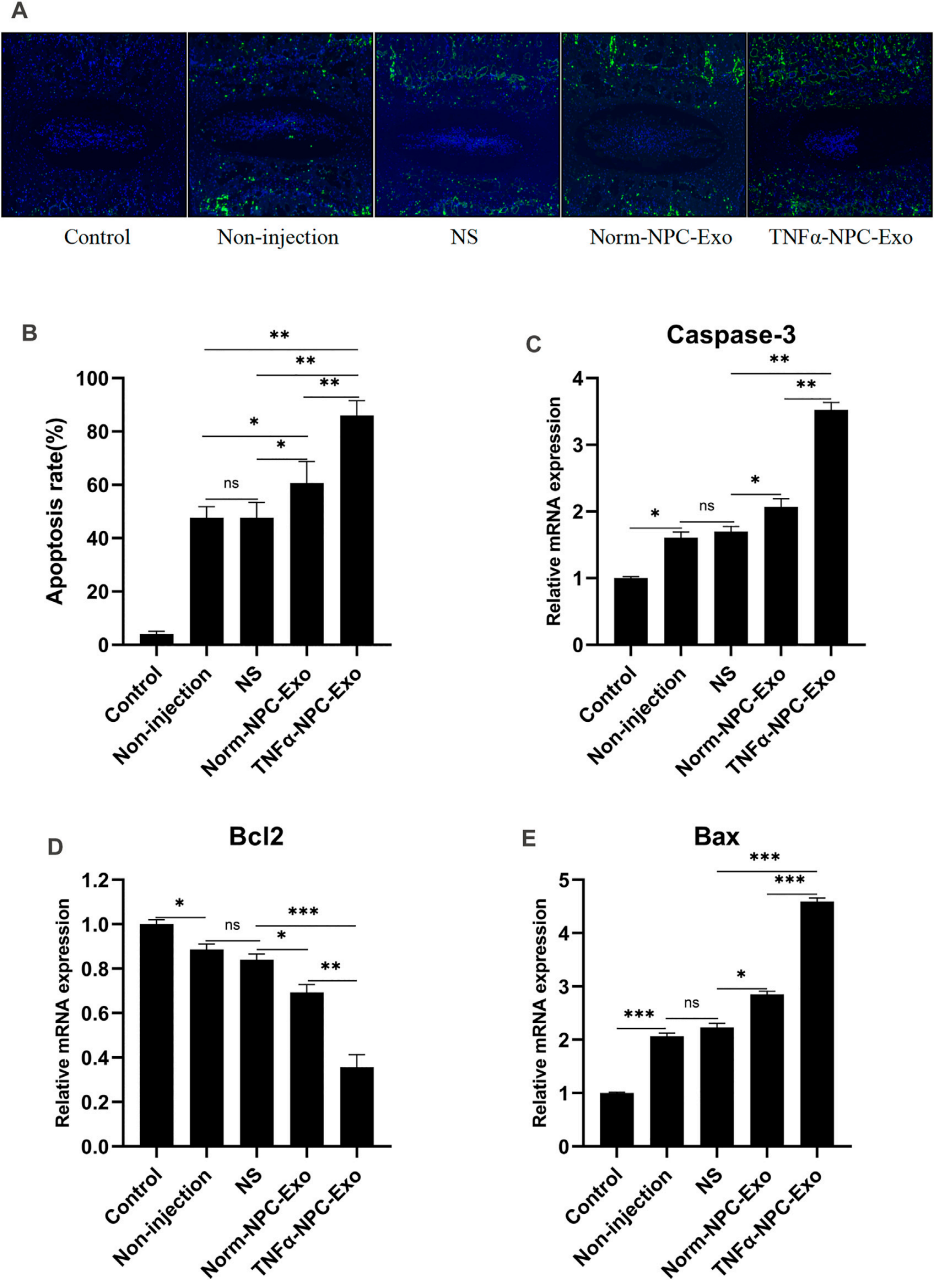
Intradiscal injection of TNFα-NPC-Exo promoted the CEPC apoptosis in a rat model. **(A)** TUNEL staining of IVDs in the indicated groups at 6 weeks after needle puncture. Green fluorescence (FITC) indicating TUNEL-positive cells. Blue fluorescence (DAPI) indicates total cells (scale bar = 150 μm). **(B)** A significant increase in the apoptosis rate was observed in the TNFα-NPC-Exo group compared with the non-injection group. **(C)** qRT-PCR showed that there are significantly increased levels of caspase-3 in the punctured IVDs by the injection of TNFα-NPC-Exo compared with other groups. **(D)** The mRNA expression of Bcl-2 in CEPCs was detected using qRT-PCR. Bcl-2 expression was significantly decreased following TNFα-NPC-Exo treatment. **(E)** The mRNA expression of Bax in CEPCs was detected using qRT-PCR. Bax expression was significantly increased following TNFα-NPC-Exo treatment. All results are representative of at least three independent experiments and each value is the mean ± s.d. of three determinations. *p < 0.05, **p < 0.01 and ***p < 0.001.

The rats were sacrificed at 6 weeks to separate spinal units containing intervertebral discs with upper and lower vertebral bodies. After removing the soft tissue from the spinal unit, we fixed and sliced it. Hematoxylin-eosin and Safranin O-Fast Green staining were performed. The results showed that the IVD demonstrated degenerated features after a puncture. The structure of the IVD was disordered and blurred boundaries or defects. At low magnification, the blue bands of CEP become thin or even disappear, and the tissue cavities become small or disappear. At high magnification, the number of chondrocytes decreased significantly, a large number of eosinophils gathered, and the vacuolar structure was disordered or disappeared. Compared TNFα-NPC-Exo group with other groups, it could be seen that damage to the disc structure was more severe (Figures 5F,G). TUNEL detection is used to detect the break of nuclear DNA in tissue cells during early apoptosis. It was noted that the number of apoptotic cells in the TNFα-NPC-Exo group was significantly higher than that in other groups (Figures 6A,B). Moreover, the expressions of Caspase-3 and Bax in the TNFα-NPC-EXO group were significantly higher than other groups, while the expressions of Bcl-2 were significantly lower than other groups by qRT-PCR analysis (Figures 6C–E).

## Discussion

The NP tissue is opalescent transparent gelatin, with high water content, elasticity, and soft texture, while the CEP tissue is thin and transparent (Adams and Roughley 2006). Therefore, NP tissue can be easily distinguished from the AF and CEP tissue when drawing materials. NP cells and CEP cells have no marker proteins. Hence, careful tissue separation during the operation ensured the reliability of subsequent cell isolation and culture. In the course of IVD degeneration, the intervention of inflammatory factors and inflammatory cascade are important factors to accelerate the degeneration process. Among them, TNFα is one of the most well-studied inflammatory factors and participates in degeneration by promoting ECM degradation in NP (Wang et al., 2014). There have also been previous studies using TNFα to induce degeneration of NP cells (Molinos et al., 2015). In the present study, TNFα was used to initiate the degenerative changes of NPCs. The results showed that TNFα had no effect on the growth of NPCs at the concentration of 5 ng/ml. But immunofluorescence staining and qRT-PCR showed that the synthesis and secretion of ECM (aggrecan and collagen Ⅱ) of NPCs were greatly reduced, and the expression level of decomposed ECM factor (MMP3) increased significantly under incubation of TNFα. This indicated that the NPCs underwent obvious degeneration induced by 5 ng/ml TNFα.

In the past 2 decades, many studies have shown that exosomes are extracellular vesicles with many important components, which play an important role in intercellular communication and regulation of cellular processes (Li et al., 2018; Zhang et al., 2018). To further understand the properties of extracellular vesicles and promote their application, how to isolate exosomes efficiently and specifically is a crucial technical link. Various exosome isolation and purification techniques currently used exosome-specific properties such as density, shape, size, and membrane marker proteins (Li et al., 2017; Shao et al., 2018; Ludwig et al., 2019). According to various comparisons of exosome separation techniques, density gradient super centrifugation is the gold standard for exosome separation, and it is also the most commonly used and reported separation technique (Zarovni et al., 2015). Further, to check and identify the quality of exosomes, internationally recognized identification methods including TEM (morphology and size of exosomes), NTA (particle size and distribution of exosomes), and western blot determination (membrane marker proteins) were used to identify exosomes, to ensure that the quality of exosomes extracted would not affect subsequent experiments. The results of our study showed that the extracted particle showed double concave disc under the electron microscope, the average particle size detected by NTA was 153.6 nm, and negative for Calnexin and positive for CD9, CD63, and TSG101 by western blot. Our results are consistent with the general characteristics of exosomes. However, the weak protein band development, combined with the results of total protein determination of exosomes, may be related to the low concentration of exosomes in NPCs. In addition, the concentration of exosomes secreted by NPCs in conventional culture was compared with that secreted by NPCs induced by TNFα. The results showed that the degenerated nucleus pulposus cells could secrete more exosomes. To sum up the above three identification results, it was proved that exosomes were obtained from the supernatant of cultured nucleus pulposus cells by density gradient super centrifugation.

The main pathologic features of IVD degeneration include inactivation and decrease in several functional cells, inflammation, decreased synthesis, and increased decomposition of ECM (Clouet et al., 2009). Thus, the integrity of the disc structure is damaged or even lost. In the past, when understanding IVD degeneration, the initiating factor of degeneration has changed from NP degeneration to CEP degeneration, which is based on the theory that the nutrition and blood supply of NPCs come from CEP (Nachemson et al., 1970; Zhu et al., 2016). Lou et al. found that cartilage endplate stem cells could inhibit intervertebral disc degeneration by releasing exosomes to nucleus pulposus cells to activate Akt/autophagy (Luo et al., 2021). However, with the increasing understanding of information exchange between cells, especially the emergence of exosomes as an important role of paracellular secretion pathway in living cells, the present study investigated the effect of degenerated NPC exosomes on the degeneration of CEP. *In vitro* experiments, firstly, it was confirmed that CEPC could take up the NPC exosomes by exosome tracer experiment. After incubating CEPC with NPC-Exo labeled with PKH67 for 24 h, a large number of exosomes were taken up by CEPC. Anatomically, the CEP is located above and below the NP, and the gap is very narrow (Raj 2008). Exosomes are essentially lipid bilayers, which can protect the material carried inside the cell outside and can be easily accessed by the cell (Simons and Raposo 2009). Hence, the NPC exosomes can easily reach the cep and be ingested. Subsequently, we examined the proliferation, apoptosis, and ECM of CEPCs under different conditions. Our study found that both Norm-NPC-Exo and TNFα-NPC-Exo had a less significant effect on the proliferation of CEPCs. In the treatment of TNFα-NPC-Exo, Caspase-3, Cleaved caspase-3, and Bax expression were significantly increased, while Bcl-2 expression was decreased. Bcl-2, Bax, and Caspase-3 are markers of cell apoptosis (D'Arcy 2019). Bcl-2 decreased, Bax and Caspase-3 increased, and the Bcl-2/Bax ratio decreased significantly, indicating that cell apoptosis occurred. TNFα-NPC-Exo could induce apoptosis of CEPCs by flow cytometry detection. In addition, it was detected by immunofluorescence that TNFα-NPC-Exo inhibits the synthesis of ECM and promotes degradation of ECM. Therefore, the degeneration of NPC exosomes could induce the degeneration of CEPCs.

The risk factors for IVD degeneration are varied and complex. Currently known risk factors include genes, injury, nutrient deficiency, abnormal mechanical load and so on (Hadjipavlou et al., 2008). The model of rat caudal vertebra by acupuncture is to induce IVD degeneration by leading to IVD inflammation through injury (Zhang et al., 2009). This method is simple in operation and has a high success rate. Radiographs showed a significant decrease in intervertebral height after a puncture on X-ray films, and disc dehydration and blackening on MRI. At the TNFα-NPC-Exo group, the above imaging changes are more obvious. Based on previous studies (Daly et al., 2016), this radiographic change indicated disc degeneration. The IVD is composed of three parts: the annulus fibrosus, the nucleus pulposus, and the cartilage endplate. By staining the slices of the rat coccyx, it can be found that once the degeneration of the IVD occurs, its structural changes are omni-dimensional, manifesting as structural disorders, blurred boundaries and even defects, reduction of functional cells, and aggregation of immune cells. These pathological changes have been similarly verified in previous studies (Wuertz and Haglund 2013; Colombier et al., 2014012). *In vivo* experiments, we further investigated the function of NPC exosomes in a rat model of IVD degeneration. Consistent with the results from *in vitro* experiments, the effects of TNFα-NPC-Exo on promotion IVD degeneration were proved by X-ray, MRI, and histology, especially, in the CEP structure damage or even defects. In addition, the TUNEL assay showed a higher apoptosis rate in the TNFα-NPC-Exo group. Previous studies have confirmed that apoptosis is one of the main pathological changes of intervertebral disc degeneration (Long et al., 2019; Cazzanelli and Wuertz-Kozak 2020; Xu et al., 2020). This suggests that TNFα-NPC-Exo aggravates IVD degeneration by promoting CEPC apoptosis. The Bcl-2 family was an important protein family that regulates apoptosis and jointly determines whether a cell entered the apoptotic process by mediating the signaling pathway of the mitochondrial pathway. Mitochondria-mediated apoptosis is mainly caused by changes in mitochondrial membrane permeability, loss of transmembrane point sites, and release of apoptosis-related factors (Tower, 2015). The Caspase family usually inactivated the cell survival pathway and specifically activates other factors that promote apoptosis (Porter and Jänicke 1999). In our experiment, the expression of Bax and Caspase-3 was significantly increased and the expression of Bcl-2 decreased in the degenerated CEP tissue. This further illustrates that TNFα-NPC-Exo promoted CEPC apoptosis. Tissue engineering is a promising strategy for IVD degeneration due to its ability to restore a healthy microenvironment and promote IVD regeneration (Dou et al., 2021). Knowledge about the understanding of the communication between NPC and CEPC is critical for tissue engineering and future clinical applications. And exosomes may be an important vector for repairing intervertebral disc degeneration.

## Conclusion

**FIGURE 7:**
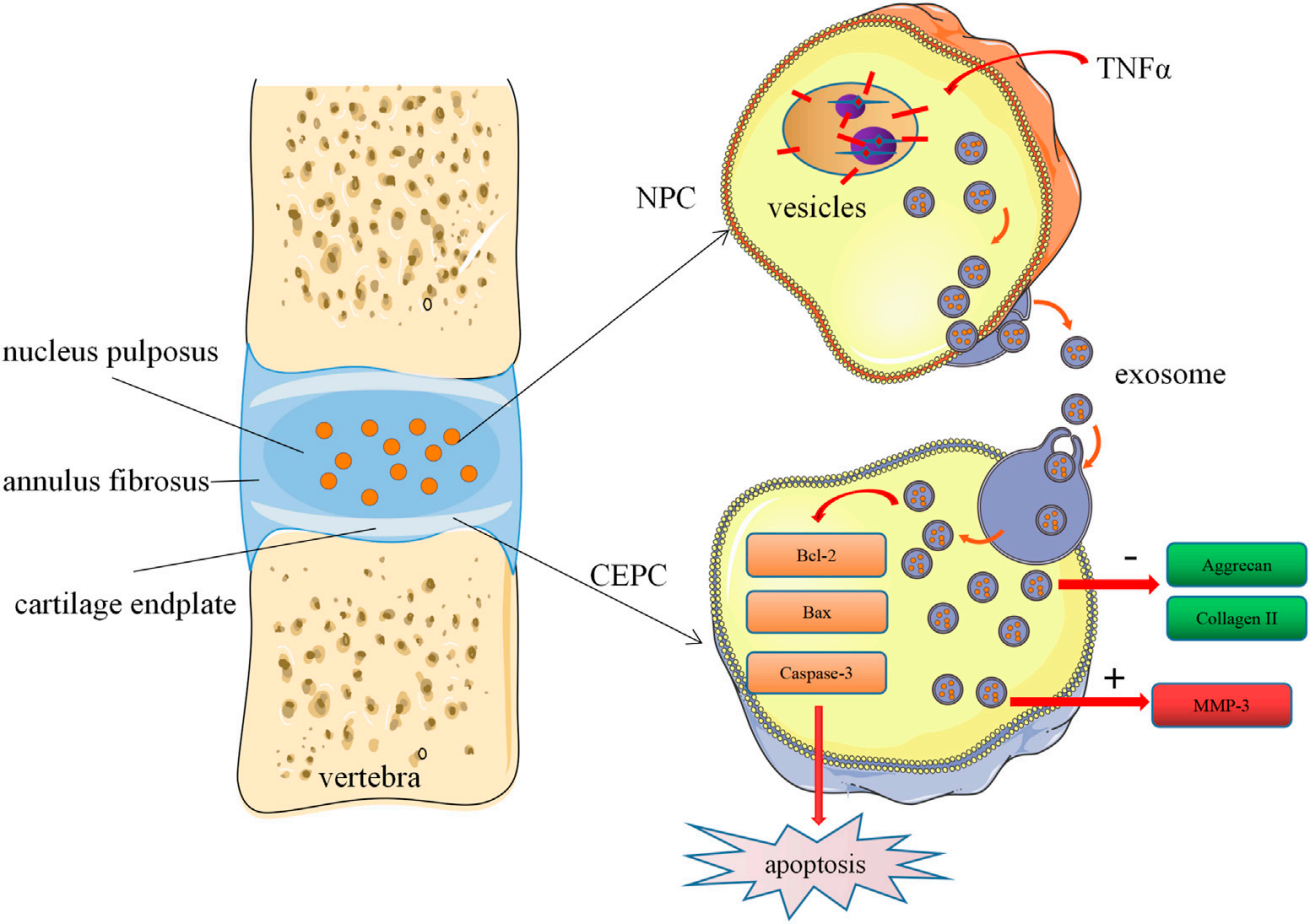
Schematic of our working hypothesis. Degenerated NPC-derived exosomes induce apoptosis of CEPCs, inhibit ECM synthesis, and promote ECM degradation.

In conclusion, our study demonstrated the cells-cells communication between degenerated nucleus pulposus cells and cartilage endplate cells. Degenerated NPC-exosome could induce apoptosis of CEPCs, inhibit the synthesis of ECM and promote degradation of ECM. Furthermore, we proved that degenerated NPC-exosome aggravates intervertebral disc degeneration (Figure 7). Our work confers a potential mechanism for IVD degeneration. However, the shortcoming of our study is that we have not been able to further investigate what substances are present in degenerated nucleus pulposus cells exosomes, which is also the direction of our future work.

## Data Availability

The datasets presented in this study can be found in online repositories. The names of the repository/repositories and accession number(s) can be found in the article/Supplementary Material.
