# Supplementary material for: Degenerative Nucleus Pulposus Cells Derived Exosomes Promoted Cartilage Endplate Cells Apoptosis and Aggravated Intervertebral Disc Degeneration
Source: Front Mol Biosci. 2022 Mar 14;9:835976. doi: 10.3389/fmolb.2022.835976 (PMC8963919; doi:10.3389/fmolb.2022.835976)
Supplement: Supplementary file 1 [file DataSheet1.PDF]

Table 1 Primer sequences

| Gene ID | Primer sequences（5’- 3’） | |
|---|---|---|
| Aggrecan | Forward Primer | CCACTGGAGAGGACTGCGTAG |
| | Reverse Primer | GGTCTGTGCAAGTGATTCGAG |
| Collagen 2A1 | Forward Primer | TGGACGATCAGGCGAAACC |
| | Reverse Primer | GCTGCGGATGCTCTCAATCT |
| MMP-3 | Forward Primer | GGTTCTCCATTCCTTTGATGGGGGGAAAGA |
| | Reverse Primer | CTTCCTGGAATTCACATCACTGCCACCACT |
| GAPDH | Forward Primer | TGTGGGCATCAATGGATTTGG |
| | Reverse Primer | ACACCATGTATTCCGGGTCAAT |
| Caspase-3 | Forward Primer | ATGGACAACAACGAAACCTC |
| | Reverse Primer | TTAGTGATAAAAGTACAGTTCT |
| Bcl-2 | Forward Primer | CAGTTGGGCAACAGAGAACCAT |
| | Reverse Primer | AGCCCTTGTCCCCAATTTGGAA |
| Bax | Forward Primer | CGAGTGGCAGCTGACATGTTTT |
| | Reverse Primer | TGAGGCAGGTGAATCGCTTGAA |

Table 2. Classification of Disc Degeneration

| Grade | Structure | Distinction of Nucleus and Anulus | Signal Intensity | Height of Intervertebral Disc |
|---|---|---|---|---|
| I | Homogeneous, bright white | Clear | Hyperintense,isointense to cerebrospinal fluid | Normal |
| II | Inhomogeneous with or without horizontal bands | Clear | Hyperintense,isointense to cerebrospinal flfluid | Normal |
| III | Inhomogeneous, gray | Unclear | Intermediate | Normal to slightly decreased |
| IV | Inhomogeneous, gray to black | Lost | Intermediate to hypointense | Normal to moderately decreased |
| V | Inhomogeneous, black | Lost | Hypointense | Collapsed disc space |
